# A simple enzymatic assay for the quantification of C1-specific cellulose oxidation by lytic polysaccharide monooxygenases

**DOI:** 10.1007/s10529-019-02760-9

**Published:** 2019-11-20

**Authors:** M. B. Keller, C. Felby, C. A. Labate, V. O. A. Pellegrini, P. Higasi, R. K. Singh, I. Polikarpov, B. M. Blossom

**Affiliations:** 1 Department of Geosciences and Natural Resource Management, University of Copenhagen, Copenhagen, Denmark; 2 Luiz de Queiroz College of Agriculture, University of São Paulo, Piracicaba, Brazil; 3 São Carlos Institute of Physics, University of São Paulo, São Carlos, Brazil; 4 Department of Chemistry, University of Copenhagen, Copenhagen, Denmark

**Keywords:** Lytic polysaccharide monooxygenase (LPMO), Biomass degradation, Quantification assay, LPMO inactivation, Cellulose oxidation, Gluconic acid

## Abstract

**Objective:**

The development of an enzymatic assay for the specific quantification of the C1-oxidation product, i.e. gluconic acid of cellulose active lytic polysaccharide monooxygenases (LPMOs).

**Results:**

In combination with a β-glucosidase, the spectrophotometrical assay can reliably quantify the specific C1- oxidation product of LPMOs acting on cellulose. It is applicable for a pure cellulose model substrate as well as lignocellulosic biomass. The enzymatic assay compares well with the quantification performed by HPAEC-PAD. In addition, we show that simple boiling is not sufficient to inactivate LPMOs and we suggest to apply a metal chelator in addition to boiling or to drastically increase pH for proper inactivation.

**Conclusions:**

We conclude that the versatility of this simple enzymatic assay makes it useful in a wide range of experiments in basic and applied LPMO research and without the need for expensive instrumentation, e.g. HPAEC-PAD.

## Introduction

Since their discovery, Lytic polysaccharide monooxygenases (LPMOs) have received considerable interest, due to their ability to boost the degradation of lignocellulose (Vaaje-Kolstad et al. 2010; Horn et al. 2012). LPMOs are mono-copper enzymes with the active site positioned on a relatively flat surface enabling them to interact with crystalline polysaccharides. The catalytic mechanism of LPMOs involves the reduction of the copper from Cu(II) to Cu(I) by an external reducing agent. Hereafter, the copper-active site of the LPMO reacts with either O_2_ or H_2_O_2_ (Walton and Davies 2016; Bissaro et al. 2017) and binds an activated oxygen species that enables the oxidation of the polysaccharide substrate. The oxidative cleavage of the β-1,4 glycosidic bonds can occur at the C1 or the C4 position or both, generating oxidized products, both soluble and insoluble (Quinlan et al. 2011). The soluble products are commonly detected by anion exchange chromatography coupled to a pulsed amperometric detector (HPAEC-PAD) (Westereng et al. 2013). It offers high sensitivity and provides detailed information about the product pattern but its limitations are the absence of commercially available reference compounds, time-consuming sample analysis, and costly instrumentation.

LPMO assays have been developed to circumvent some of these challenges. The Amplex red assay provides a fast measure of LPMO reactivity and is based on the release of H_2_O_2_ in the absence of a substrate (Kittl et al. 2012). Recently, a spectrophotometrical assay was developed that exploits peroxidase activity of some LPMOs to oxidize 2,6-dimethoxyphenol (Breslmayr et al. 2018). While both assays can be employed to detect LPMO reactivity, the results cannot be directly correlated to the oxidative activity of LPMOs on polysaccharides. Soluble LPMO products have been analyzed with by carbohydrate gel electrophoresis (PACE) (Quinlan et al. 2011; Frandsen et al. 2016) yielding semi-quantitative results. LPMO activity has also been analyzed using chromogenic polysaccharide hydrogel substrates (Kračun et al. 2015). Assays for quantifying C1-oxidized moieties in the insoluble fraction of the LPMO on pure cellulose model substrates were described, such as a fluorescence-based approach (Vuong et al. 2017), and a colorimetric cation-based assay (Wang et al. 2018). Despite the value of these methods, LPMO activity assays and especially methods that can simplify the quantification of LPMO oxidation, without the necessity of expensive specialized instrumentation, not only on cellulosic model substrates but on complex substrates, such as lignocellulosic biomass are still lacking.

We describe a simple quantification method for cellulose active C1-oxidizing LPMOs, employing a commercially available glycoside hydrolase to hydrolyze the oxidation products to yield gluconic acid and glucose. Subsequently, gluconic acid is quantified spectrophotometrically which is suitable for microplate formats. The release of C1-oxidized products by *Tt*LPMO9E on microcrystalline cellulose (Avicel) and steam pretreated wheat straw (PWS) was quantified and compared to the results by HPEAC-PAD.

## Methods

### Substrates

The activity of *Thielavia terrestris* LPMO9E (*Tt*LPMO9E) (previously *Tt*GH61E) was determined on the two substrates; Avicel PH101 (Sigma-Aldrich), and pretreated wheat straw (PWS). The wheat straw was hydrothermally pretreated at 190 °C for 10 min at an initial solid loading of 35%. The heating was done by injection of steam, decreasing the solids level to 15% after 10 min treatment. Using standard NREL methods the composition was determined to be 54% glucan, 4% xylan, 34% lignin, and 6% ash. The substrates were washed five times in MilliQ water and twice in 50 mM citrate buffer pH 6 to remove soluble sugars prior to the incubation with *Tt*LPMO9E.

### Enzymes

*Tt*LPMO9E was donated by Novozymes A/S (Bagsværd, Denmark). The *Tt*LPMO9E concentration was determined by absorbance at 280 nm using a molar extinction coefficient of 58,120 M^−1^ cm^−1^. Novozym 188 was obtained from Novozymes A/S (Bagsværd, Denmark). β-glucosidase from *Aspergillus niger* and cellobiohydrolase I (CBHI) from *Trichoderma longibrachiatum* were purchased from Megazyme with a specified activity of 40 U ml^−1^ and a concentration of 10 mg ml^−1^, respectively.

### Preparation of C1-oxidized cello-oligosaccharide standards for the HPAEC-PAD

C1-oxidized cello-oligosaccharide standards for the HPAEC-PAD were prepared by oxidizing pure cello-oligosaccharides (Megazyme) with iodine as earlier described for the preparation of cellobionic acid (Green et al. 1976; Hildebrand et al. 2016), with the following modifications:

The cello-oligosaccharide was dissolved in a minimum of deionized water and heated at 55 °C for 10 min until dissolved. A solution of iodine in methanol was added to a final molar ratio of 1:2:110 (cello-oligosaccharide:iodine:methanol) and the solution was heated at 40 °C for 15 min. At room temperature 4% (w/v) potassium hydroxide-methanol solution was slowly added to the solution and left for an additional 30 min to a final molar ratio of 1:12 (cello-oligosaccharide:potassium hydroxide). 20 mL deionized water was added to the solution and the methanol was gently boiled off in a rotary evaporator. The pH of the solution was set to pH 5 with citric acid.

All procedures were done with constant stirring.

### Inactivation of *Tt*LPMO9E

Samples of Avicel 50 g L^−1^, 50 mM citrate buffer pH 6, 1 µM *Tt*LPMO, and 2 mM ascorbic acid in a final volume of 200 µl were mixed in Eppendorf tubes. The samples were treated with or without 1 mM EDTA addition, with or without 50 mM NaOH and with or without incubation in a water bath at 100 °C for 20 min. A positive control, not treated with EDTA, NaOH, or heat, was included. The samples were incubated in an Eppendorf ThermoMixer C at 50 °C for 6 h at 1100 rpm. Subsequently, the samples were centrifuged and the supernatant was kept for measuring C1-oxidized sites as described below.

### *Tt*LPMO9E activity

Samples of Avicel (50 g L^−1^) or PWS (20 g L^−1^), 1 µM *Tt*LPMO9E, 2 mM ascorbic acid in 50 mM citrate buffer pH 6 in a final volume of 200 µl were incubated in an Eppendorf ThermoMixer C at 50 °C. At varying time points (0–24 h), the enzymatic reaction was quenched by the addition of NaOH to a final concentration of 50 mM.

After 24 h, the samples were centrifuged at 1800×*g* and the supernatant was used for measuring C1-oxidized sites as described below.

The pellet was further hydrolyzed for measuring C1-oxidized sites in the insoluble fraction. The pellet was washed five times in 200 µL 50 mM citrate buffer pH 5, by centrifugation and resuspension, to decrease pH and remove soluble sugars. The pellet was resuspended in 50 mM citrate buffer. Cellobiohydrolase I (CBHI) from *Trichoderma longibrachiatum* was added to a final concentration of 0.5 g L^−1^ in a final volume of 200 µL. The samples were incubated in an Eppendorf ThermoMixer C at 50 °C for 24 h at 1100 rpm after which the samples were centrifuged. The supernatant was used for measuring C1-oxidized sites revealed by the CBHI as described below.

### Quantification of gluconic acid by HPAEC-PAD

100 µL of the supernatant was diluted in 50 mM citrate buffer pH 5 together with 5 U ml^−1^ β-glucosidase from *Aspergillus niger* to a final volume of 200 µL. The samples were incubated at 50 °C for 24 h before the samples were filtered through a nylon filter with a pore size of 0.45 µm and loaded in HPLC vials.

The samples were analyzed by high-performance anion-exchange chromatography (HPAEC) performed on an ICS5000 system, equipped with a pulsed amperometric detector (PAD) (Thermo Scientific) with an analytical CarboPac PA1 column (2 × 250) as described elsewhere (Westereng et al. 2013) with some modifications: The analytes were eluted at 0.25 mL min^−1^ at 30 °C and the initial conditions were 100% eluent A (0.1 M NaOH). A linear gradient was applied increasing the proportion of eluent B (1 M NaOAc in 0.1 M NaOH) to 90% A:10% B after 10 min. An exponential gradient (curve 6) was applied to reach 83.1% A:16.9% B after 22 min and 0% A:100% B after 23 min. These conditions were kept for 4 min. The column was reconditioned by running initial conditions for 15 min. Gluconic acid was quantified based on external standards of 0–250 mg L^−1^ gluconic acid in 50 mM citrate pH 5.

### Quantification of gluconic acid by the d-Gluconic acid/d-Glucono-δ-lactone assay kit

Gluconic acid was quantified using the assay kit from Megazyme (Bergmeyer and Moellering 1988). The method is based on phosphorylation of gluconic acid to D-gluconate-6-phosphate by ATP in the presence of gluconate kinase with the simultaneous formation of ADP. In the presence of NADP, gluconate-6-phosphate is oxidatively decarboxylated by 6-phosphogluconate dehydrogenase to ribulose-5-phosphate with the stoichiometrically formation of NADPH. The assay was performed according to the microplate procedure described by the manufacturer with the following modifications: 50 µL of the supernatant was mixed with 160 µL MilliQ water, 20 µL solution I (buffer), 20 µL solution II (NADP + ATP), 6 µL solution III (6-PGDH) diluted three times in MilliQ water. The samples were mixed in an Eppendorf ThermoMixer for 4 min at 600 rpm after which the absorbance was measured at 340 mm in microplate reader (Molecular Devices SpectraMax i3). 6 µL solution IV (GCK) diluted three times with MilliQ water was added to the samples and the plate was mixed in an Eppendorf ThermoMixer at 600 rpm for 6 min. The absorbance was measured at 340 nm and the difference was used to quantify gluconic acid based on external standards of 0–250 mg L^−1^ gluconic acid in 50 mM citrate pH 5. According to the supplier’s information, the range of linearity of the UV-signal is 0.1–5 µg gluconic acid per sample in a sample volume of 1–200 µL. Using the guidelines of the International Conference on Harmonisation (ICH), we calculated the quantitation limit (LOQ) as LOQ = 10Sa/b, where Sa is the standard error of the y-intercept of the regression line of the gluconic acid standard curve and b is the slope of the regression line of the gluconic acid standard curve. Using this approach, we found that LOQ = 0.27 mg gluconic acid L^−1^.

### Quantification of glucose

The hydrolysis yield was determined on an Ultimate 3000 high-performance liquid chromatography equipped with an ultraviolet detector at 210 nm (Thermo Scientific). The separation was performed on a Phenomenex Rezex ROA column at 80 °C with a mobile phase of 5 mM H_2_SO_4_ at a flow rate of 0.6 mL min^−1^. The glucose was quantified based on external standards of 0–10 g L^−1^ glucose in 50 mM citrate pH 5.

## Results and discussion

### Hydrolysis of oxidized products

**Fig. 1:**
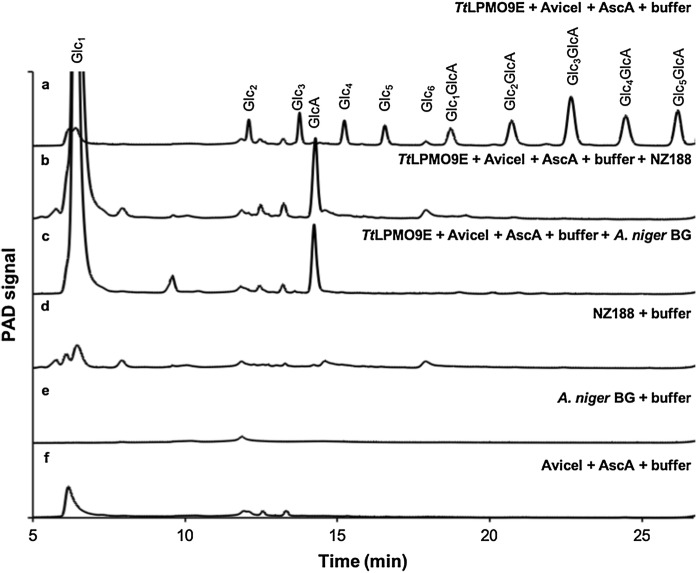
HPAEC-PAD chromatograms and elution patterns. **a**, The supernatant of 50 g L^−1^ Avicel incubated with 1 µM *Tt*LPMO9E and 2 mM ascorbic acid in 50 mM citrate buffer pH 6 for 6 h at 50 °C. The peaks are assigned based on external standards of C1-oxidized cello-oligosaccharide standards (not shown). The oligosaccharides are abbreviated Glc_N_ and Glc_N_GlcA with N being the number of glucose units and GlcA being gluconic acid. **b**, The supernatant from **a** incubated with Novozym 188. **c**, The supernatant from **a** incubated with *A. niger* β-glucosidase. **d**, Novozym 188. **e**, *A. niger* β-glucosidase. **f** The supernatant of 50 g L^−1^ Avicel incubated with 2 mM ascorbic acid for 6 h at 50 °C

To decrease product complexity, the products of LPMO-catalysis are often hydrolyzed by glycoside hydrolases prior to the quantification by HPAEC-PAD (Cannella et al. 2016; Bissaro et al. 2017; Frommhagen et al. 2017). Here, we assessed the applicability of two commercially available β-glucosidases, Novozym 188 and β-glucosidase from *Aspergillus niger* to hydrolyze the soluble products generated by *Tt*LPMO9E from a cellulosic substrate to the monomers, glucose and gluconic acid (Fig. 1).

The lowest β-glucosidase concentration capable of hydrolyzing the *Tt*LPMO9E products generated in the following experiments to glucose and gluconic acid was determined to be 1.25 U/ml *A. niger* β-glucosidase corresponding to a specific activity on 4-Nitrophenyl β-D-glucuronide of 1.26 × 10^−3^ µmol p-nitrophenol min^−1^ and Novozym 188 corresponding to a specific activity of 1.16 × 10^−3^ µmol p-nitrophenol min^−1^ according to the β-glucosidase activity assay described elsewhere (Gunata et al. 1990). These concentrations were used throughout the work presented here. The product profiles were analyzed by HPAEC-PAD (Fig. 1).

Both β-glucosidases hydrolyzed the *Tt*LPMO9E products to the monomers, glucose and gluconic acid (GlcA). As the Novozym 188 contained impurities eluting together with glucose and gluconic acid, the *A. niger* β-glucosidase was selected for further use.

### Inactivation of *Tt*LPMO9E

As *Tt*LPMO9E does not have activity on soluble oligosaccharides (Westereng et al. 2015), the reaction in the supernatant can simply be stopped by filtration. Efficient inactivation of the LPMOs is, however, essential to quantify the activity in the insoluble fraction. Prior to the hydrolysis of the LPMO products by glucoside hydrolases, common procedures to quench the LPMO reaction are 10 min incubations at 100 °C (Cannella et al. 2016; Frommhagen et al. 2017) or by increasing pH (Isaksen et al. 2014; Bissaro et al. 2017). Previous studies have shown that chelating agents, such as EDTA can remove the copper atom from the LPMO active site (Vaaje-Kolstad et al. 2010; Kracher et al. 2017), suggesting that the addition of EDTA could effectively inactivate LPMOs.

**Fig. 2:**
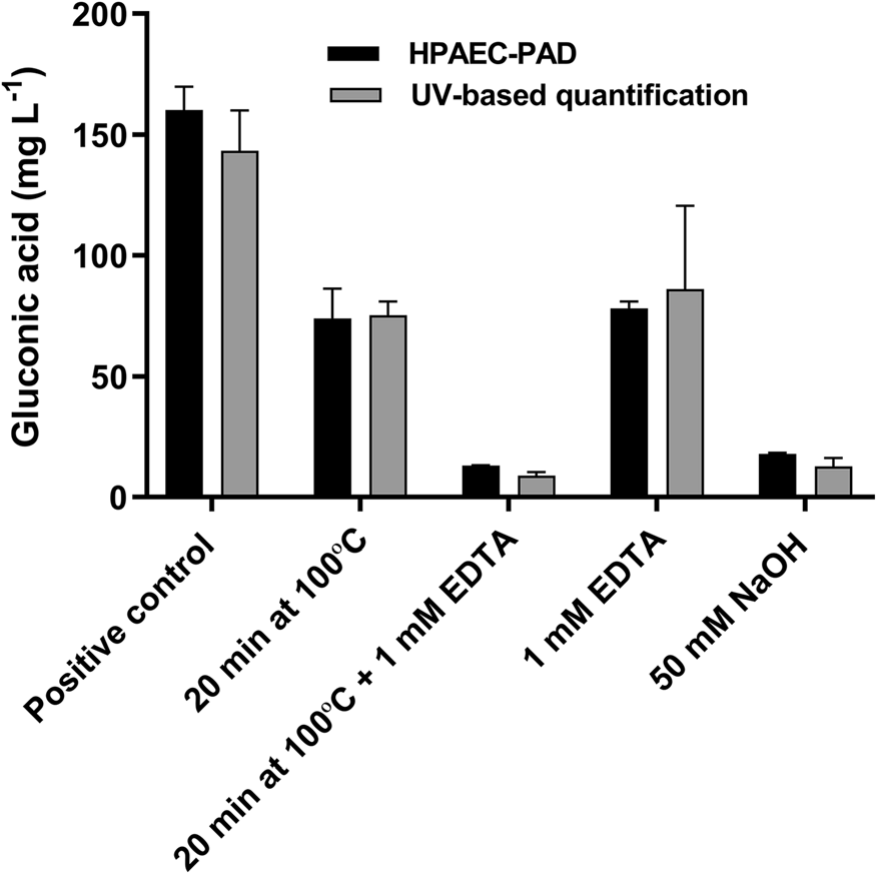
Evaluation of methods to inactivate *Tt*LPMO9E by quantification of gluconic acid. 50 g L^−1^ Avicel were incubated with 2 mM ascorbic acid and 1 µM *Tt*LPMO9E in 50 mM citrate buffer pH 6 for 6 h at 50 °C. Prior to the incubation, the samples were treated with either, 20 min incubation at 100 °C, 20 min incubation at 100 °C + 1 mM EDTA, 1 mM EDTA, or 50 mM NaOH. A positive control, not treated with NaOH, EDTA, or incubation at 100 °C, was included. Gluconic acid was quantified by HPAEC-PAD (black bars) and by the UV-based gluconic acid kit (grey bars). All incubations were performed in triplicate and the standard deviations of the samples are represented by error bars

The inactivation efficiencies were tested by measuring the activity of *Tt*LPMO9E after boiling for 20 min with and without the addition of EDTA or after the addition of sodium hydroxide, and their activity was compared to the activity of a non-treated *Tt*LPMO9E samples (Fig. 2).

*Tt*LPMO9E retained approximately 50% of its activity after boiling for 20 min. This may be due to reversible thermal unfolding as has previously been reported for *Nc*LPMO9C and *Ta*LPMO9A, respectively (Kracher et al. 2017; Singh et al. 2019). These findings suggest that if boiling is used to attempt inactivation of LPMOs, it should be followed by careful evaluation of residual activity. The addition of EDTA decreased the activity by approximately 50%. A combination of boiling and addition of EDTA decreased the activity of *Tt*LPMO9E to less than 10% compared to the positive control not treated with heat or EDTA. A similar decrease in activity was obtained by the addition of 50 mM NaOH to reach pH 11.

### Quantification of the LPMO reaction

**Fig. 3:**
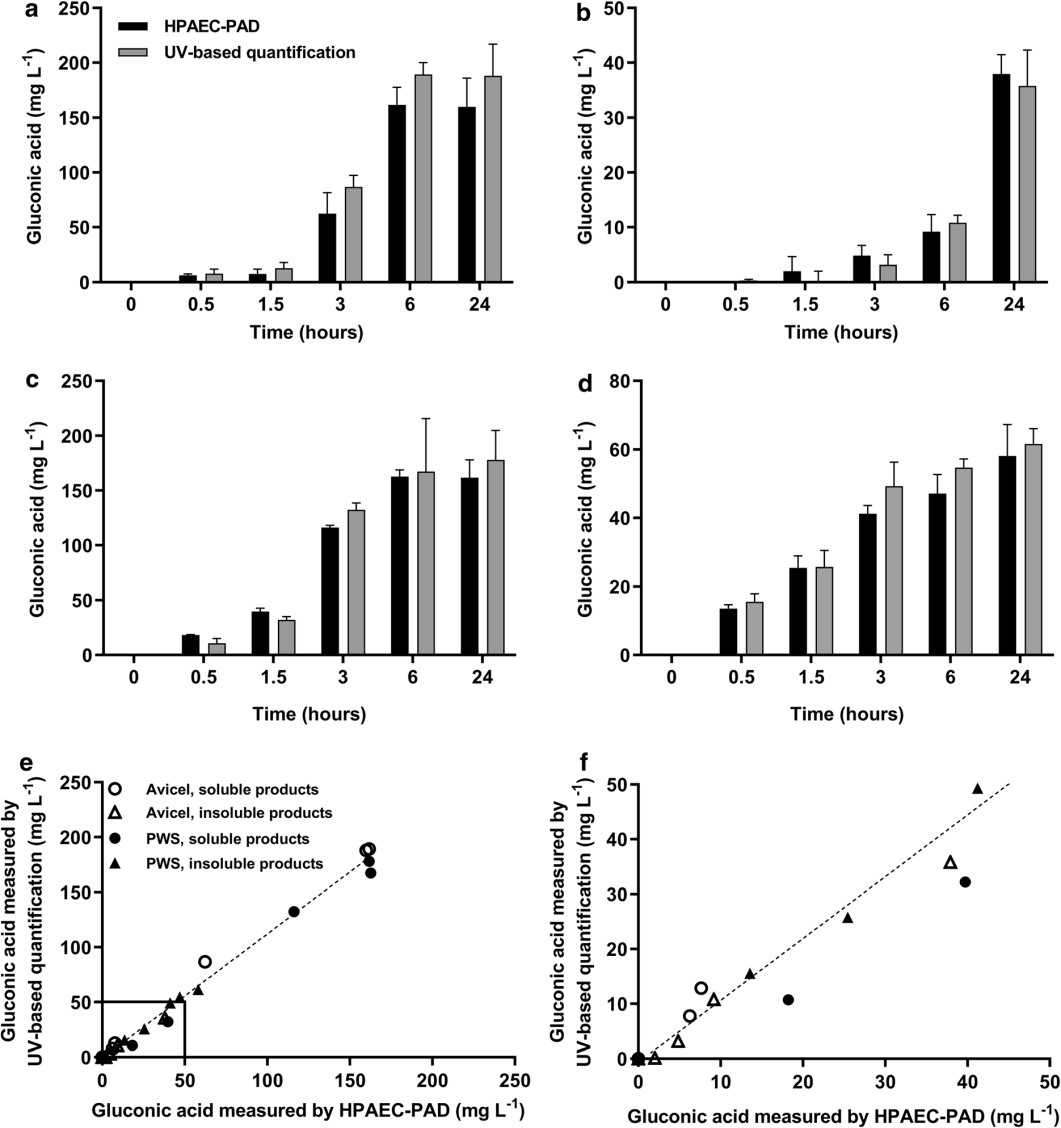
Quantification of the soluble and insoluble products from microcrystalline cellulose and pretreated wheat straw (PWS). 50 g L^−1^ Avicel or 20 g L^−1^ PWS incubated with 2 mM ascorbic acid and 1 µM *Tt*LPMO9E in 50 mM citrate buffer pH 6 at 50 °C from 0 to 24 h. **a**, Supernatant of Avicel incubated with *Tt*LPMO9E and ascorbic acid incubated with 5 U mL^−1^*A. niger* β-glucosidase. **b**, Pellet of Avicel incubated with *Tt*LPMO9E and ascorbic acid incubated with 0.5 g L^−1^ T*. longibrachiatum* CBHI and 5 U mL^−1^*A. niger* β-glucosidase. **c**, Supernatant of PWS incubated with *Tt*LPMO9E and ascorbic acid incubated with 5 U mL^−1^*A. niger* β-glucosidase. **d**, Pellet of PWS incubated with *Tt*LPMO9E and ascorbic acid incubated with 0.5 g L^−1^ T*. longibrachiatum* CBHI and 5 U mL^−1^*A. niger* β-glucosidase. Gluconic acid detected after 0 h of incubation was subtracted from the results. Gluconic acid was quantified by HPAEC-PAD (black bars) and by the UV-based gluconic acid kit (grey bars). All results are averages of triplicate measurements. The standard deviations of the samples are represented by error bars. **e** and **f**, Correlation between the quantification results measured by HPAEC-PAD (y-axis) and gluconic acid kit (x-axis) from **a**–**d**. The equation for the best linear regression (slope = 0.9) and the coefficient of determination, r^2^ (r^2^ = 0.98) is shown. **f**: Enlargement of the correlation at gluconic acid concentrations from 0 to 50 mg L^−1^. For clarity, standard deviations are not shown

The activity of *Tt*LPMO9E was measured by quantifying the C1-oxidation products in the soluble and the insoluble fraction of microcrystalline cellulose (Avicel) and pretreated wheat straw (PWS) (Fig. 3). The results should not be interpreted as the absolute quantification of insoluble products, as only a fraction of the insoluble products was hydrolyzed.

The formation of gluconic acid (GlcA) in the soluble fraction of Avicel incubated with *Tt*LPMO9E and ascorbic acid increased up to approximately 175 mg L^−1^ after 0.5 to 6 h. Between 6 and 24 h, no further changes in GlcA concentration were detected in the soluble fraction. GlcA formation in the insoluble fraction of Avicel increased up to approximately 35 mg L^−1^ after 24 h. Interestingly, after 6 h of incubation an increase in GlcA was solely detected in the insoluble fraction whereas no increase in GlcA was detected in the soluble fraction.

For the reactions on pretreated wheat straw, the GlcA content in the soluble fraction increased from 0.5 to 6 h up to approximately 175 mg L^−1^. As with the reaction on Avicel, the GlcA concentration in the soluble fraction remained unchanged between 6 and 24 h. In the insoluble fraction, the formation of GlcA increased steadily up to approximately 60 mg L^−1^ after 24 h.

The UV-based enzymatic GlcA quantification method produced similar results and detection limits as the HPAEC-PAD when analyzing products from the soluble and insoluble fractions of Avicel as well as PWS. The results of the two methods showed a linear correlation with r^2^ values within the four data groups varying between 0.986 and 0.996.

**Fig. 4:**
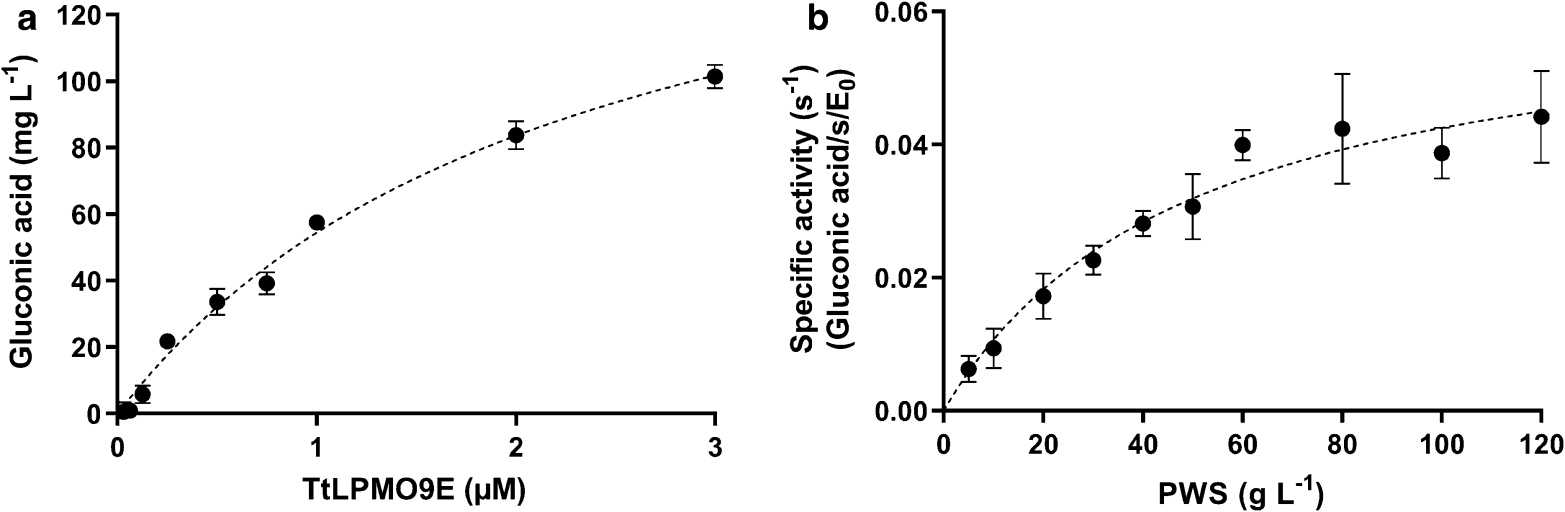
Dose–response curves of enzyme and substrate titrations. **a**, Quantification of the soluble products of 20 g L^−1^ PWS incubated with 2 mM ascorbic acid and varying concentrations of *Tt*LPMO9E in 50 mM citrate buffer pH 6 at 50 °C for 3 h. The dotted line represents the best fit of a hyperbolic function. **b**, specific activity of *Tt*LPMO9E on PWS measured by quantifying the gluconic acid in the soluble products of varying loads of PWS incubated with 2 mM ascorbic acid and 0.25 µM *Tt*LPMO9E in 50 mM citrate buffer pH 6 at 50 °C in 3 h. The dotted line represents the best fit of a hyperbolic function. All results are averages of triplicate measurements. The standard deviations of the samples are represented by error bars

Furthermore, dose–response curves of enzyme titration (Fig. 4a) and substrate loading (Fig. 4b) were performed on PWS and the results were fitted to a hyperbolic function with the best fit of [y = 197.97*×/(2.51 + x)] for the enzyme titration data. Likewise, the dose–response curve with increased substrate load was fitted to a hyperbolic Michalies-Menten like function,

$$_{p} V_{ss} = \frac{{_{p} V_{\max } S_{0} }}{{_{p} K_{M} + S_{0} }}$$

as described for other interfacial enzymes (Cruys-Bagger et al. 2013) with _p_V_ss_ being the specific activity (product/time/enzyme concentration), _p_V_max_ being the maximal specific activity, S_0_ being the substrate load, and _p_K_M_ the Michaelis–Menten constant. Using the enzymatic assay, the _p_K_M_ and the _p_V_max_/E_0_ were derived from the regression analysis to be 49.80 g L^−1^ and 0.064 s ^−1^ (3.8 min ^−1^), respectively. The ladder being in line with the results given in a recent review, comparing the apparent enzyme rates of LPMOs, that showed that the apparent catalytic rates (k_cat_) of LPMOs fall in a relatively small range roughly between 1 and 10 min ^−1^ when not considering H_2_O_2_ or light-driven reactions (Bissaro et al. 2018).

## Conclusions

**Fig. 5:**
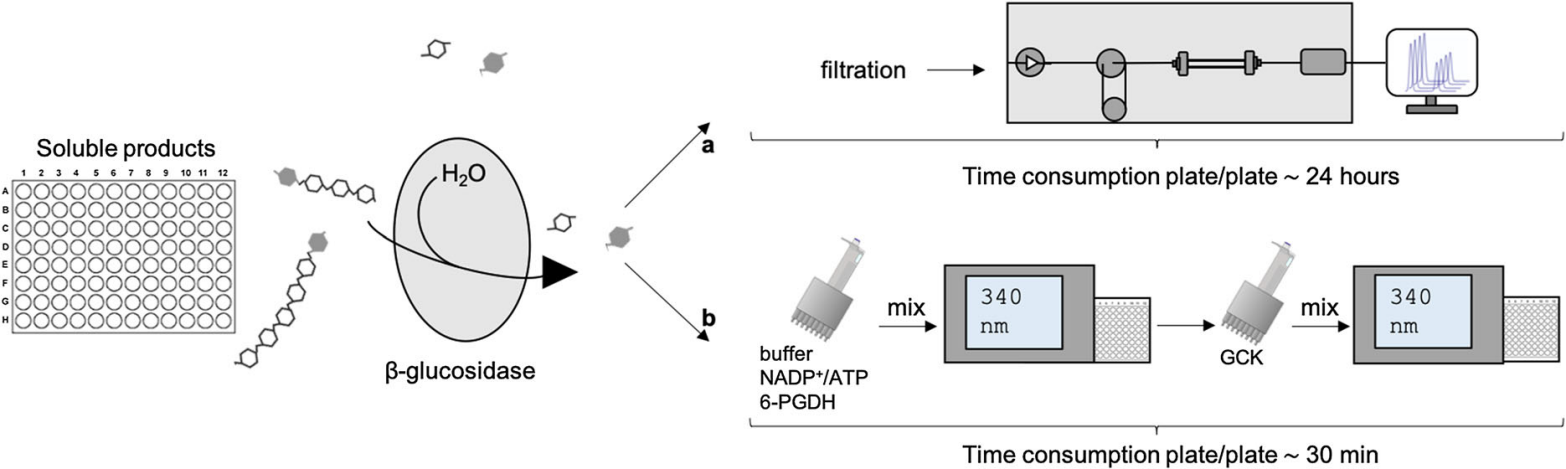
Schematic representation of the UV-based enzymatic assay to quantify LPMO activity on cellulose. After the LPMO reaction, the plates were centrifuged and the soluble products were collected in the supernatant. β-glucosidase was added to the soluble products and the plates were incubated at 50 °C overnight after which gluconic acid was quantified by HPAEC-PAD (**a**) and by the UV-based enzymatic assay (**b**). **a**: The samples were filtered and gluconic acid was quantified on a CarboPac PA1 column (2 × 250) on a HPAEC-PAD. The peak areas were used for the quantification of gluconic acid based on external standards. The time consumption was 30 min per plate, however this could be optimized for shorter runs. **b**: Buffer, NADPH + , and 6-Phosphoglucanate dehydrogenase was added to the samples. The samples were mixed for 4 min in a thermomixer at 600 rpm and the absorbance was measured at 340 nm after which gluconate kinase was added to the samples and the plates were mixed for 6 min in a thermomixer at 600 rpm and the absorbance was measured at 340 nm. The difference in absorbance was used for the quantification of gluconic acid based on external standards

We have described a simple, fast, and sensitive UV-based enzymatic assay to quantify specifically the C1-oxidation of cellulose active LPMOs. In recent years, a number of LPMO assays have been developed to decrease the analysis time compared to HPAEC-PAD, as well as eliminating the need for oxidized oligosaccharide standards that are not commercially available, and expensive instrumentation. These methods all have distinct advantages making them suitable for different applications. The method reported in this paper excels by its simplicity and its ability to quantify soluble and insoluble LPMO products on a pure cellulose substrate as well as on a lignocellulosic biomass. The UV-based enzymatic assay is suitable for an auto-analyzer format and, with an analysis time of approximately 30 min per 96 samples in a microtiter plate, compared to approximately 15 min per sample or 24 h per 96 samples in a microtiter plate with HPAEC, the method offers fast measurements (Fig. 5).

Furthermore, we compared methods for *Tt*LPMO9E inactivation and demonstrated that a boiling is not sufficient. Thus, we suggest to apply a metal chelator while boiling or to drastically increase pH for proper inactivation.

The quantification method may be useful in other experiments, such as for comparison of activities of different C1-oxidizing cellulose active LPMOs, for studies of LPMO activities on different substrates and for testing compounds that might influence LPMO activity. Such experiments are technically straightforward by using the quantification approach outlined here, but changing conditions should be followed by calibration against an alternative quantification technique to exclude potential interferences.
